# Optimal-robust selection of a fuel surrogate for homogeneous charge compression ignition modeling

**DOI:** 10.1371/journal.pone.0234963

**Published:** 2020-06-25

**Authors:** Irene García-Camacha Gutiérrez, Raúl Martín Martín, Josep Sanz Argent

**Affiliations:** 1 Institute of Applied Mathematics for Science and Engineering (IMACI), University of Castilla-La Mancha, Toledo, Spain; 2 Fundación Valenciaport, Valencia, Spain; George Mason University, UNITED STATES

## Abstract

Homogeneous Charge Compression Ignition (HCCI) combustion is a potential candidate for dealing with the stringent regulations on vehicle emissions while still providing very good energy efficiency. Despite the promising results obtained in preliminary studies, the lack of autoignition control has delayed its launch in the engine industry. In the development of the HCCI concept, the availability of reliable computer models has proved extremely valuable, due to their flexibility and lower cost compared with experiments using real engines. In order to obtain the best formulation of a fuel surrogate formulated with n-heptane, toluene and cyclohexane that efficiently estimate the autoignition behaviour, regression adjustments are made to the Root-Mean-Square Errors (RMSE) of experimental Starts of Combustion (SOC) from the modeled SOC. The canonical form of the Scheffé polynomials is widely used to fit the data from mixture experiments, however the experimenter might have only partial knowledge. In this paper we present the adaptation of the robust methodology for possibly misspecified blending model and an algorithm to obtain tailor-made optimal designs for mixture experiments, instead of using standard designs which are indiscriminately employed, to make good estimations of the parameters blending model. We maximize the determinant of the mean squared error matrix of the least square estimator over a realistic neighbourhood of the fitted regression mixture model. The maximized determinant is then minimized over the class of possible designs, yielding an optimal design. Thus, the computed desings are robust to the exact form of the true blending model. Standard mixture designs, as the simplex lattice, are around 25% efficient for estimation purposes compared with the designs obtained in this work when deviances from the considered model occur during the experiments. Once an optimal-robust design was selected (based on the level of certainty about model adequacy), we computed the optimal mixture that best reproduces the combustion property to be imitated. Optimal mixtures obtained when the considered model is inadequate agree with the results achieved in empirical studies, which validates the methodology proposed in this work.

## Introduction

Governments and other institutional entities have shown their concern about the release of hazardous substances from the use of Internal Combustion Engines in transportation, and the resulting impact on human health. Consequently, they have made the regulations on pollutant emissions stricter. On the other hand, there is also global awareness regarding the efficient use of fossil fuels due to their proven impact on global warming. Thus, new methods of combustion need to be explored to generate cleaner energies achieving the best performance. Whilst other alternative methods, such as electric engines or the use of fuel cells, are being developed, internal combustion engines are still the most widely used nowadays. Engine researchers and designers look into improvements in traditional Spark Ignition (SI) and Compression Ignition (CI) engines, to reduce their emissions while maintaining good fuel economy. Homogeneous Charge Compression Ignition (HCCI) combustion is a potential candidate to meet these needs and has gathered extensive support over the last decade.

HCCI combustion is defined as a process by which a homogeneous mixture of air and fuel is compressed until autoignition occurs near the end of the compression stroke [1]. HCCI engines have the advantage of providing good fuel economy, like diesel, while producing very low particulate emissions, comparable to a gasoline engine. A comparative study of the different combustion engines can be found in [1].

One of the main features of this type of engine is that combustion is a kinetically controlled process rather than a physical phenomenon as in traditional engines [2]. Autoignition and combustion rates are strongly influenced by the chemical kinetics of the fuel. These phenomena are therefore extremely sensitive to charge composition as well as other factors involved in the combustion such as pressure and temperature. Charge composition is the main scenario of this work and different levels of pressure and temperature are considered through the parameter combinations used in the experiments. On the other hand, ignition timing is the major challenge to be addressed in these engines. This is the main drawback which has delayed its launch on the automobile market. Different solutions have been considered, such as the variable valve timing (VVT) method or the variable EGR method among others (see, for example, [3–5]). However, all these strategies have been studied experimentally. Mathematical engine models are valuable tools for predicting and analyzing these processes and allow many engine design alternatives to be considered [6]. Due to the paramount importance of the combustion kinetics of the fuel, any type of model (from zero dimensional to CFD models) will necessarily require reliable methods for accurately describing the reaction kinetics of real fuels. Because of the great complexity of commercial fuels (which include from hundred to thousands of different hydrocarbons) surrogate fuels are used instead, which are mixtures of a few hydrocarbons with well-known combustion kinetics. This paper poses the problem of fuel surrogate selection to control HCCI autoignition from an Optimal Experimental Design (OED) perspective, and incorporates the theory of robust design into the problem, to consider a more realistic scenario.

Surrogates must closely mimic market fuel properties and match engine combustion and emissions behavior [7]. A variety of selection mechanisms have been used in the literature. Hernández et al. [8] assumed that the error function matching the simulated and experimental starts of combustion had an absolute minimum, and sought this optimum using a type of bisection method. Others, however, defined an objective function representing some physical, chemical and combustion properties and optimized this complex function using commercial software optimizers [9–11] or parallel computing calculation [12]. Yu et al. [13], took a different approach, constructing surrogate fuel mixtures by directly matching the molecular structure and the key functional groups, instead of using the targets for the combustion properties explicitly. There are as many fuel surrogates as engine properties to be imitated. In the particular case of HCCI modeling and the present research, the surrogate should adequately represent both the autoignition properties and commercial fuel compositions. Classical mixture experimental designs have been used to estimate the effects of fuel compositions to HCCI performance [14]- [15]. Their applicability depends however on the experimental factor space and on the choice of a model for the responses. The novelty of this work is that, in addition to consider the optimal design theory, considers possible departures from the assumed model. It is a common situation in this type of experimental setups due to the extreme conditions under which experiments are carried out.

Computer simulation has become a dominant tool in making HCCI a reality and in the quest for control strategies for HCCI, and has higher flexibility and lower cost than real engine experiments [6].There has been increasing interest in Optimal Experimental Design (OED) in recent year, not only for reasons of resource optimization but also due to its flexibility in handling more complex and realistic problems. Applications of this theory to engines include, for example, the modeling of the ignition angle in HCCI engines [16] or the quantification of the effects of fuel compositions on emissions [17]. On the other hand, in this type of problem, the response of the model may be easily perturbed by the extreme conditions under which experiments are carried out. Practitioners have little information about model suitability prior to running the experiments and the large number of factors involved in the combustion as well as the difficulty of considering all possible operating conditions implies that small deviations from the considered model may occur during the experiments. The robust design theory allows practitioners to establish the level of certainty about model suitability. Thus, this approach would reflect a more realistic modeling of the problem. Taking these considerations into account, the aims of this research are:

- to formulate the problem of selecting a fuel surrogate to model HCCI autoignition using optimal mixture design.
- to incorporate robustness into the formulation.
- to provide a new method to obtain an efficient *D*−optimal robust design for several levels of certainty about model suitability.
- from the selected *D*−optimal robust design, to compute the surrogate formulation that best reproduces the mechanism to be replaced as well as the combustion property to be imitated.

These objectives are aimed at selecting a fuel surrogate to estimate the autoignition time under real HCCI conditions.

## Materials and methods

The strong dependency of HCCI autoignition on the chemical kinetics of the fuel implies that it is extremely sensitive to charge composition. Commercial diesel consists of hundreds of medium-high molecular weight hydrocarbons, and thus it is not feasible to consider the oxidation chemistry of all the compounds when modeling targets [8]. A strategy commonly adopted in simulation studies is to consider a reduced number of species, and then to prove that the reduced mechanism properly matches the desired combustion property through experimentation. Following Hernández et al. [8], our interest is in modeling the Root-Mean-Square Error (RMSE)

$$\begin{array}{c} \text{RMSE}=\sqrt{\frac{\sum_{i=1}^{J}{\left({\text{SOC}}_{i}\left(\text{exp}\right)-{\text{SOC}}_{i}\left(\text{mod}\right)\right)}^{2}}{J}}, \end{array}$$ (1)

where SOC_*i*_(exp) and SOC_*i*_(mod) correspond to the experimental and modeled Starts Of Combustion (SOC) obtained in *J* engine tests. In particular, SOC reports the crank angle corresponding to 10% of the cumulative heat released during the high temperature combustion process. Eq (1) defines the response variable in the model. For notational reasons, this will be represented by $\text{RMSE}\underset{Not}{\equiv }Y$. Experimental SOCs were obtained using a single-cylinder diesel engine operating under HCCI conditions while the modeled values came from the zero-dimensional single-zone kinetic model implemented in the CHEMKIN 4.0 software [18].

Single-cylinder engine tests provide a means for excellent control and reproducibility of the operating conditions [19]. The engine used for the experimental work was a four-stroke single cylinder engine, with 0.287l swept volume and four valves, second generation common rail (fuel injection system), 14:1 compression ration, 0.068m x 0.079m (bore x stroke), and 12 x 86 *μ*m number and diameter of injector holes respectively. Different engine operating conditions were considered in the *J* engine tests run in (1) modifying the most important parameters affecting the two-stage oxidation kinetics of any fuel. The charge composition was quantified by the exhaust gas recirculation percentage (EGR). The engine load was quantified in terms of the indicated mean effective pressure (IMEP) by fixing the intake pressure and varying the EGR rate. The engine speed was measured in revolutions per minute (rpm), and the start of injection (SOI) was defined with respect to the top dead center (TDC) where a negative value indicates that injection starts before TDC. Operating rate data are shown in Table 1. As it can be observed, experimental HCCI conditions were achieved with a very early fuel injection. Consequently, the fuel/air mixture may assume to be homogeneous (or almost homogeneous) from the end of the injection process, since the physical delay can be considered negligible compared to the chemical one [8]. In addition, the injection pressure was kept constant for all the tests at 900 bar.

**Table 1:** Engine operating conditions used in the engine test.

Engine speed (rpm)	EGR rate (%)	SOI (with respect to TDC)	IMEP (bar)
1500	40	-80	5.2
			2.8
		-60	6.3
			4.1
	60	-80	4.9
			2.8
		-60	6.2
			4.1
2000	40	-80	5.9
			3.1
		-60	5.8
			4.4
	60	-80	5.6
			3.2
		-60	7.1
			4.6

Simulations were performed using CHEMKIN code, which assumes uniform temperature, pressure and species concentration in the combustion chamber, and ignores the temperature differences near boundary zones due to heat transfer. A single zone model was used. In spite of the limitations of these models due to the assumption that the composition and temperature in the cylinder are homogeneous, they have been used to provide an estimation of the ignition timing [20–22]. As previously mentioned, another fundamental characteristic of the selected surrogate is that it must appropriately reproduce the kinetic-chemical mechanism of the original fuel. The affinity criterion means that the main hydrocarbon families must be represented in the mixture. Since original diesel consists of around 37% paraffins, 34% naphthenes and 29% aromatics by mass [8], *n*−heptane, cyclohexane and toluene were the selected components to represent each hydrocarbon family. *n*−heptane has a cetane number equal to diesel fuel and its oxidation has been widely validated under engine conditions [23–25]. Kinetic models of longer paraffins show similar autoignition results to *n*−heptane and, for this reason, some authors recommend the use of *n*−heptane for its lower computational cost [26]. The cyclohexane and toluene were selected since their oxidation chemistry is validated in the literature under similar conditions to HCCI engines [27–32]. The surrogate mechanism was built up in a step-wise fashion, using as a starting point the *n*−heptane detailed mechanism proposed by Curran et al. [33] which contains 550 species and 2450 reactions. Then, the reactions that describe the oxidation of toluene proposed by the Lawrence Livermore National Laboratory [27] were added (285 species and 1427 reactions). In addition, the 12 co-oxidation reactions between *n*−heptane and toluene proposed by Andrae et al. [34] were incorporated into the mechanism. Finally, the mechanism of cyclohexane oxidation, containing 1081 species and 4269 reactions, given by Silke et al. [32] were added. The resulting mechanism contains 1140 species and 4578 reactions.

A suitable fit of the response requires an appropriate choice of both design and model. Since the RMSE strongly depends on the considered proportions of *n*−heptane, toluene and cyclohexane and these are ingredients of a mixture, models for mixture experiments may be appropriate for explaining this behavior. Controlled variables in a standard mixture problem are non-negative, belonging to [0, 1] and dependent through the relationship $1_{q}^{\prime }p=1$ where $1_{q}={\left(1,\ldots ,1\right)}^{\prime }\in R^{q}$ and ***p*** = (*p*_1_, …, *p*_*q*_)′ is the vector of relative proportions in a *q*−component mixture. These constraints define the design region as a (*q*−1)−dimensional simplex $S=\{p\in {\left[0,1\right]}^{q}\;:1_{q}^{\prime }p=1\}$. A mixture design *ξ* is thus an allocation rule of experimental units over S,

$$\begin{array}{c} \xi ={\left\{\begin{array}{c} p\\ m(p) \end{array}\right\}}_{p\in S}, \end{array}$$

where *m*(***p***) is a probability mass function. If *m*(***p***) is finite-supported, it is said that *ξ* is a discrete design. Otherwise, it is a continuous design. As stated above, a suitable model must be selected a priori to describe the composition-response relationship. Scheffé polynomials are widely applied to fit the response surface. With regard to polynomial order, quadratic models have been used in HCCI modeling as they provide a reasonable balance between the necessary number of experiments and modeling capacity [16]. The general form of a second-degree mixture model is:

$$E\left[Y\left(p\right)\right]=\theta_{0}+\sum_{j=1}^{q}\theta_{j}p_{j}+\sum_{i=1}^{q-1}\sum_{j=1}^{q}\theta_{ij}p_{i}p_{j},$$

where the term $\sum_{j=1}^{q}\theta_{j}p_{j}$ is the linear blending portion of the model, while the term $\sum_{i=1}^{q-1}\sum_{j=1}^{q}\theta_{ij}p_{i}p_{j}$ represents the nonlinear blending (curvature) between component pairs. However, ordinary polynomials do not allow the parameters to be uniquely identified, due to co-linearity between proportions. Instead, canonical polynomials introduced by Scheffé [35], [36] are the most commonly used in mixture experiments for a large number of practical situations, including HCCI combustion properties ([14]- [15], [37]). Thus, a canonical second-order Scheffé polynomial in the intercept form (2) was considered for modeling the RMSE

$$\begin{array}{c} Y\left(p\right)=f^{\prime }\left(p\right)\theta +\varepsilon =\theta_{0}+\theta_{1}p_{1}+\theta_{2}p_{2}+\theta_{11}{p_{1}}^{2}+\theta_{22}{p_{2}}^{2}+\theta_{12}p_{1}p_{2}+\varepsilon , \end{array}$$ (2)

where *p*_1_, *p*_2_ represent the proportion of *n*−heptane and toluene by mass respectively and the proportion of cyclohexane in mass, *p*_3_, is implicitly determined by the relationship *p*_3_ = 1−*p*_1_−*p*_2_. The vector ***θ*** = (*θ*_0_, *θ*_1_, *θ*_2_, *θ*_11_, *θ*_22_, *θ*_12_) corresponds to the vector of the unknown model parameters. Additive uncorrelated random errors *ε* with common variance will be assumed.

Classical optimal design theory establishes that the model form is completely determined, and then, the so-called optimal designs are obtained from the optimality criteria based on the inverse of the Fisher information matrix (*M*^−1^(*ξ*)). J. Sanz [37] stated that there are significant discrepancies between experimental and modeled results despite the existence of validated mechanisms in the literature to reproduce HCCI engine conditions. The large number of factors involved in this process, as well as the difficulty of considering all possible operating conditions, implies that small deviations from the model may occur during the experiments. Thus the standard models could not accomodate other effects of the mixture components. Robust design theory introduced by Huber [38] and later developed by Wiens [39–41] will be suitably applied to address this situation. A more realistic framework of the problem is to consider a class of plausible responses

$$\begin{array}{c} E\left(Y|p\right)=f{}^{\prime }\left(p\right)\theta +\psi \left(p\right),\;\;p\in S, \end{array}$$ (3)

where *ψ* is a *contamination function* which belongs to a certain class of functions Ψ. The choice of Ψ determines the neighborhood of possible responses. This study will consider a neighborhood orthogonal to the experimenter’s assumed response, since it covers a wide range of general alternative responses (for further details see [41]). When the assumed response is (2) but the true one is (3), bias is introduced in the least square estimator (*lse*) $\hat{\theta }$ of the model parameters. Let *n* be the number of mixtures to perform. Then, unlike in classical optimal design theory, the mean-square-error matrix of the estimators, rather than the variance, is a good measure of design quality

$$\begin{array}{c} \text{mse}\left(\hat{\theta }\right)=\text{mse}\left(\psi ,\xi \right)=\frac{\sigma_{\varepsilon }^{2}}{n}M^{-1}(\xi )+M^{-1}(\xi )b(\psi ,\xi )b^{\prime }(\psi ,\xi )M^{-1}(\xi ) \end{array}$$ (4)

where

$$\begin{array}{c} M(\xi )=\int_{S}f\left(p\right)f^{\prime }\left(p\right)m\left(p\right)dp\;\text{and}\;b(\psi ,\xi )=\int_{S}f\left(p\right)\psi \left(p\right)m\left(p\right)dp. \end{array}$$

The first term in (4) corresponds to the variance-covariance matrix of $\hat{\theta }$ assuming homoscedastic errors and common variance $\sigma_{\varepsilon }^{2}$, while the second is the squared bias. Suitable functionals of (4) are defined to optimize some aspect of the model. Classical alphabetical optimality criteria [42] define the most common optimal robust criteria in terms of (4). In this context, they are known as *loss functions* L(ψ,ξ). The *D*−optimality criterion minimizes the volume of the confidence ellipsoid of the model parameter estimators. Since it has a direct interpretation and considers all model parameters, this criterion has became the most popular among practitioners and will be used in this research. Thus, our objective is to obtain the *minimax* design

$$\begin{array}{c} \xi^{*}=\arg\;\underset{\xi \in \Xi }{min}\;\underset{\psi \in \Psi }{max}L_{D}\left(\psi ,\xi \right), \end{array}$$

where *Ξ* is the set of probability measures. In other words, the *D*−optimal robust design will be the design that minimizes the loss in the worst scenario. The maximization problem over Ψ may be equivalently formulated as a problem of computing the maximum eigenvalue ch_max_ of a certain matrix [39]. Therefore, the *D*−loss is defined as

$$\begin{array}{ccc} L_{D}\left(\xi \right) & = & \left(1-\nu \right){\left(\frac{1+\frac{\nu }{1-\nu }\;{\text{ch}}_{\text{max}}\;M^{-1}\left(\xi \right)G\left(\xi \right)}{\text{det}M(\xi )}\right)}^{1/k}, \end{array}$$ (5)

where

$$\begin{array}{c} G(\xi )=K(\xi )-H(\xi ),\;K(\xi )=\int_{S}f\left(p\right)f^{\prime }\left(p\right)m^{2}(p)dp,\;H(\xi )=M(\xi )A^{-1}M(\xi ), \end{array}$$

*k* is the number of model parameters, $A=\int_{S}f\left(p\right)f^{\prime }\left(p\right)dp$ and the parameter *ν* has been defined from the neighborhood ratio *τ* and the variance as

$$\begin{array}{c} \nu =\frac{\tau^{2}}{\sigma_{\varepsilon }+\tau^{2}}. \end{array}$$

This new parameter represents the relative importance, to the experimenter, of the errors due to bias rather than to variance. The 0 value means that the model parameters are unbiased, while 1 represents the opposite case.

To clarify the importance of the designs created by this approach, we define the efficiency of a design *ξ* with respect to the design $\bar{\xi }$

$$\begin{array}{c} \text{Eff}\left(\xi \right)={\left(\frac{L_{D}\left(\xi \right)}{L_{D}\left(\bar{\xi }\right)}\right)}^{\frac{1}{k}}\times 100. \end{array}$$

If a design *ξ* has, for instance, 70% efficiency with respect to $\bar{\xi }$, then $\bar{\xi }$ will produce the same precision as *ξ* with 30% fewer observations, thus reducing the experimental and resource cost.

Since a finite number of trials must be proposed to carry out the simulations, *n*−point discrete designs will be considered. This implies that prior definitions must be properly discretized. Then, for each fixed *ν*, the optimization problem to solve is

$$\begin{array}{c} \xi^{*}=\arg\underset{\xi \in \Xi }{min}\;\left(1-\nu \right){\left(\frac{1+\frac{\nu }{1-\nu }\;{\text{ch}}_{\text{max}}\;M^{-1}\left(\xi \right)G\left(\xi \right)}{\text{det}M(\xi )}\right)}^{1/p}. \end{array}$$

In most cases, this problem is analytically intractable. A genetic algorithm (GA) with specialized operators has been developed in this study for this purpose. The algorithm starts by generating a population of *M* random initial designs. A *generation* corresponds to a complete renovation of the population. This update is carried out through the *genetic operators*. They are classified into three groups: *selection, crossover and mutation operators*. Two *parent* designs are suitably selected and an *offspring* design is created from them by the action of the operators. A measure of probability (*fitness*) is assigned to each design. This assignment is made according to design goodness measured in terms of criterion function values. Selection operators always act until they have completed a generation while reproduction and mutation operators only act if a probability test is passed. A random *U*(0, 1) is generated in each possible intervention and if this value is lower than a certain predetermined probability, the operator will act. Otherwise, the design will remain unchanged. The reproduction probability chosen in this study was *P*_*C*_ = 0.9 while the mutation probability *P*_*Mut*_ was automatically updated to generate more opportunities for new solutions when the algorithm is further from the optimum, and to reduce the “biodiversity” otherwise. It is noteworthy that the appropriate selection of the operators considered in this paper, as well as the creation of new ones because of the special nature of mixture experiments, converged for all study cases. Pseudo-code for the proposed GA is given in the S1 Appendix. It was implemented in MATLAB R2018b and the code is available from the authors.

## Results

**Fig 1:**
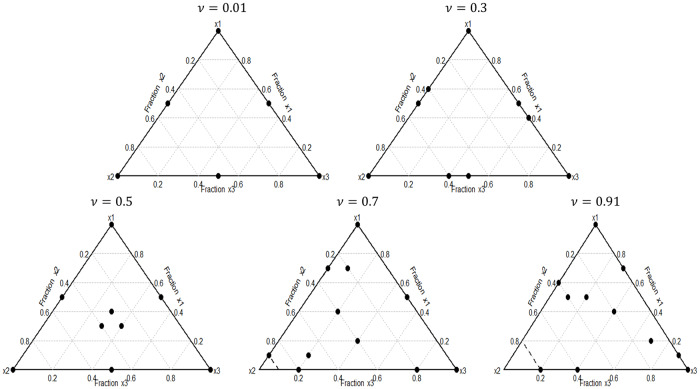
*D*−optimal robust designs. Designs obtained with the proposed GA for HCCI autoignition modeling through model (2).

Several numerical examples were run covering different levels of certainty about model suitability (*ν*). *n* = 12−point designs were considered for illustration. The *D*−optimal robust designs obtained are shown in Fig 1. They were graphically depicted using the *mixexp* R package. In these graphs, simplex vertices represent pure components, edges correspond to binary blends and simplex interior points contain all three-component systems.

As one might expect, quasi-zero *ν* value design was supported on the extremes and mid-edges of the design region according to classical *D*−optimal design. The class of so-called *standard mixture designs* was obtained in this study case. If *m* ≥ 1 is an integer, the {*q*, *m*}−simplex lattice on S is defined as the set of points whose coordinates are integer multiples of 1/*m*, that is $\left\{p\in S,\;p_{i}=\frac{j}{m},0\leq j\leq m,\;1\leq i\leq q\right\}$. Thus, a {*q*, *m*}−simplex lattice design is a design with support points in the {*q*, *m*}−lattice. Another class of standard mixture designs are the {*q*, *m*}−simplex centroid designs (1 ≤ *m* ≤ *q*), which are defined as a collection of points in S with *q*−*j* coordinates equal to zero and *j* coordinates equal to $\frac{1}{j}$, *j* = 1, …, *m*. According to the previous definitions, a {3, 2}−simplex lattice design was obtained for *ν* = 0.01 and quite similar for 0.3, that is support points are pure components or binary blends; whereas a quasi {3, 3}-simplex centroid design was obtained in the case of *ν* = 0.5. No pattern is deduced from the spatial distribution of design points when the bias is emphasized (*ν* > 0.5). As we can see from Fig 1, the optimal design points are spread out over the experimental region. It is a logical result since, in order to seek protection against deviations from the model, no design region should be favored over any other. This shows the importance of considering robust designs when we cannot guarantee the model choice. The number of iterations needed to reach the optimum, as well as the loss values of the designs calculated are shown in Table 2. Note that the more the bias is ignored, the higher the loss and the computational cost.

**Table 2:** Computational costs and loss values of the *D*−optimal robust designs obtained from Fig 1.

*ν*	no. it	minloss
0.01	2567	25.42
0.3	2300	17.49
0.5	1771	11.3
0.7	1803	1.99
0.91	1161	0.6782

Since standard mixture designs such as simplex lattice or simplex centroid are the most widely used by practitioners to estimate the unknown parameters, we compute their efficiencies with respect to the designs obtained through the proposed methodology when deviations from the assumed model occur. The efficiency is a number between 0 and 100 which measures the goodness of a design *ξ* for estimating purposes. Thus, a {3, 2}−simplex lattice is 40% efficient regarding the design shown in Fig 1 when *ν* = 0.7, whereas a {3, 3}−simplex centroid is 25% efficient in this case. A more extreme study case is when *ν* = 0.91. In this context, the efficiencies are 28% and 20% according to a {3, 2}−simplex lattice and a {3, 3}−simplex centroid respectively with respect to the design shown in Fig 1 for *ν* = 0.91.

Once a *D*−optimal robust design is selected, the next step consists of appropriately fitting the model 2. The response in this model explains the error between the start of combustion obtained experimentally and simulated via the surrogate (1). The optimal choice of the fuel surrogate will be one that minimizes the response in a complex feasible region.

Taking into account the designs depicted in Fig (1), the least-squares method was used to fit the (2) with the data obtained after displaying the experimental setup described in the *Material and Method Section*. Data used to perform the adjustment are displayed in S1 Table. Thus, for different levels of certainty about model suitability, we obtain

$$\hat{Y}\left(p\right)={\hat{\theta }}_{0}+{\hat{\theta }}_{1}p_{1}+{\hat{\theta }}_{2}p_{2}+{\hat{\theta }}_{11}{p_{1}}^{2}+{\hat{\theta }}_{22}{p_{2}}^{2}+{\hat{\theta }}_{12}p_{1}p_{2},$$

where the third-component proportion is implicitly determined through the relationship *p*_3_ = 1−*p*_1_−*p*_2_. According to the similarity criterion, the optimal choice of the surrogate for this experimental setup will be the solution of the optimization problem

$$(p_{1}^{*},p_{2}^{*})=\text{arg}\underset{\begin{array}{c} \hat{Y}(p)\geq 0\\ 0\leq p_{1},p_{2}\leq 1\\ p_{1}+p_{2}\leq 1 \end{array}}{min}{\hat{\theta }}_{0}+{\hat{\theta }}_{1}p_{1}+{\hat{\theta }}_{2}p_{2}+{\hat{\theta }}_{11}p_{1}{}^{2}+{\hat{\theta }}_{22}p_{2}{}^{2}+{\hat{\theta }}_{12}p_{1}p_{2},$$ (6)

where $p_{3}^{*}=1-p_{1}^{*}-p_{2}^{*}$. Using a nonlinear optimizer in MATLAB with linear constraints, we obtained the optimal mixture compositions of the fuel surrogate that best reproduce HCCI autoignition depending on the degree of model suitability. Optimal mixtures (given in % in mass) as well as fitted models are shown in Table 3.

**Table 3:** Fitted models for optimal-robust designs shown in Fig 1 and optimal mixtures obtained from solving the optimization problem (3).

*ν*	${\hat{\theta }}_{0}$	${\hat{\theta }}_{1}$	${\hat{\theta }}_{2}$	${\hat{\theta }}_{11}$	${\hat{\theta }}_{22}$	${\hat{\theta }}_{12}$	%Â *n*−heptane	% toluene	% cyclohexane
0.01	38.04	-80.97	-66.64	46.51	54.55	53.73	72	25	3
0.3	37.74	-83.64	-42.43	49.55	30.98	34.42	75	25	0
0.5	37.26	-71.34	-56.9	36.86	44.8	43.98	80	20	0
0.7	36.58	-74.58	-37.46	40.91	24.85	33.91	83	17	0
0.91	41.18	-80.92	-5.47	42.07	-24.82	-4.44	70	30	0

## Discussion

HCCI combustion is a potential candidate for dealing with the more stringent regulations on pollutant emissions from motor vehicles, as well as the increasing concern about the emission of carbon dioxide from fossil fuels and its impact on global warming. Despite the promising results obtained in preliminary studies [1], the lack of autoignition control has delayed its launch in the engine industry. Further research is required to address this challenge. Computer simulation, however, has become a dominant tool in making HCCI a reality and in the quest for control strategies for HCCI. In addition, it has higher flexibility and lower cost compared with real engine experiments [6]. The main objective of this paper is to select a fuel surrogate capable of reproducing the autoignition characteristics of commercial fuels as well as fuel properties. To achieve this, we first provide a methodology to optimally and robustly select the simulations to obtain the best inferences of the parameters when a class of plausible responses (3) may be used to model the RMSE (2). Once the model is fitted, the optimal choice of the surrogate is obtained from solving the optimization problem (6).

Most experiments are performed in order to develop and optimize the most adequate engine control strategies do not use the tools of optimal experimental design. Consequently, data analysis will be informative only if data are themselves. Even if this methodology is applied, departures of the considered model may occur during the experimental stage invalidating, thus, the collected information in the observations. It may be due to extreme conditions under which the experiments are carried out or due to the lack of knowledge about model suitability. Practitioners have little information about model prior to running the experiments and the large number of factors involved in the combustion, as well as the difficulty of considering all possible operating conditions, implies that small deviations from the considered model may occur in a real situation. Therefore, there is a need to use a methodology which obtains robust results against possible departures from the considered model. Using this novel idea, engine researchers and designers may optimally design their experiments depending on their level of certainty about the assumed model. The main challenge faced by this research was the complex optimization design problem to be solved. A genetic algorithm was developed especially for this purpose, which is original to the authors. We should underline that this algorithm may be used regardless of the number of components or the model considered. This methodology is, therefore, general and may be applied in other engine processes where an optimal formulation is required.

## Conclusions

To conclude, the experiments obtained with the proposed methodology enhance the efficiency for purposes of estimation. Thus, depending on the level of certainty, the quality of similar designs may be different by up to 80%. In particular, considering the optimal-robust designs shown in Fig (1) and the experimental setup of [8], the optimal formulations that best match the original fuel to simulate HCCI autoignition are shown in Table 3. According to these results, optimal mixtures obtained when bias is emphasized (*ν* ≥ 0.3) agree with the results obtained in recent empirical studies [43]. In these cases, optimal robust designs incorporate interior points of the simplex. Therefore, this idea supports the need to consider optimal-robust designs when the model form is not completely known.

## Supporting information

S1 Appendix Genetic algorithm for computing *D*−optimal robust mixture designs. (PDF)

S1 Table Data used to illustrate the results with the obtained designs through the proposed methodology. (XLSX)

S2 Table *D*−optimal robust designs obtained from Fig 1 and values of RMSE achieved during the experiments. (PDF)
