# Supplementary material for: Optimal-robust selection of a fuel surrogate for homogeneous charge compression ignition modeling
Source: PLoS One. 2020 Jun 25;15(6):e0234963. doi: 10.1371/journal.pone.0234963 (PMC7316266; doi:10.1371/journal.pone.0234963)
Supplement: S1 Appendix — (PDF) [file pone.0234963.s001.pdf]

S1 Appendix. Genetic Algorithm for computing $D-$optimal robust mixture designs.

Step 0. Generate a *Population* of $M = 40$ random designs and set $k = 0$.

Step 1. Compute $A,\ M(\xi_i^{(k)}),\ K(\xi_i^{(k)}),\ H(\xi_i^{(k)}),\ G(\xi_i^{(k)})\ \forall i = 1, ..., M$.

Step 2. Calculate the losses $l_D(\xi_i^{(k)})$ and sort them in increasing order $\xi_{i_i}^{(k)}, ..., \xi_{i_M}^{(k)}$.

Step 3. Compute the fitness $fit_j = 1 \Big/ \left[\sqrt{i_j} \cdot \sum_{k=1}^{M} \left(\frac{1}{\sqrt{i_k}}\right)\right],\ j = 1, ..., M$.

Step 4. Selection

Step 4.1 *Selection with elitism*: $N_{elite} = M \cdot P_{elite}$ designs with largest fitness are left unchanged at next generation. $P_{elite} = 0.1$ in this work.

Step 4.2 *Probabilistic selection*: Two *parents* designs are proportionally chosen to their fitness values
$i_1^* = min\{i : \sum_{s=1}^{i} fit_s \geq \zeta_1\}$ and $i_2^* = min\{i : \sum_{s=1}^{i} fit_s \geq \zeta_2\}$,
where $\zeta_1, \zeta_2 \sim U(0,1)$ are randomly generated.

Step 5. Crossover *Unitary crossover:* If $\alpha \leq P_C$ ($\alpha \sim U(0,1)$),
$child^{(k+1)} = max\{\xi_{i_1^*}^{(k)}, \xi_{i_2^*}^{(k)}\}$. The excess of probability is gradually deleted by a randomization process. Otherwise, $child^{(k+1)} = \text{best}\{\xi_{i_1^*}^{(k)}, \xi_{i_2^*}^{(k)}\}$.

Step 6. Mutation Let $P_{Mut} = P_{M_{ini}} + (P_{M_{end}} - P_{M_{ini}})\frac{invariant}{invarianttotal}$ be the mutation probability, $P_{M_{ini}} = 0.1$, $P_{M_{end}} = 0$, *invariant* the number of iterations the best population design remains unmodified and *invarianttotal* the number of iterations the best design has to remain unchanged to be considered as the optimum.
If $\beta \leq P_{Mut}$ ($\beta \sim U(0,1)$), then $child^{(k+1)} = Perm(child^{(k+1)})$.
Otherwise, $child^{(k+1)} = child^{(k+1)}$ remains unchanged.

Step 7. Repeat from **Step 4.2** until $M = 40$ new designs are generated.
Update $k = k + 1$.

Step 8. Repeat from **Step 1** until $invariant = invarianttotal$.
