# Supplementary material for: Optimal-robust selection of a fuel surrogate for homogeneous charge compression ignition modeling
Source: PLoS One. 2020 Jun 25;15(6):e0234963. doi: 10.1371/journal.pone.0234963 (PMC7316266; doi:10.1371/journal.pone.0234963)
Supplement: S2 Table — (PDF) [file pone.0234963.s003.pdf]

S2 **Table.** ***D*−optimal robust designs obtained from Fig 1 and values of RMSE achieved during the experiments.**

ν	Mixture	p_1	p_2	p_3	RMSE	ν	Mixture	p_1	p_2	p_3	RMSE
0.01	1	1	0	0	3.58	0.3	1	0	0	1	38.04
	2	0	1	0	25.94		2	0	0	1	38.04
	3	0	0	1	38.04		3	0	0.5	0.5	18.35
	4	0.5	0	0.5	9.18		4	0	0.6	0.4	29.6
	5	0	0.5	0.5	18.35		5	0	1	0	25.94
	6	0.5	0.5	0	2.93		6	0	1	0	25.94
	7	1	0	0	3.58		7	0.4	0	0.6	11.3
	8	0	1	0	25.94		8	0.5	0	0.5	9.18
	9	0	0	1	38.04		9	0.5	0.5	0	2.93
	10	0.5	0	0.5	9.18		10	0.6	0.4	0	2.17
	11	0	0.5	0.5	18.35		11	1	0	0	3.58
	12	0.5	0.5	0	2.93		12	1	0	0	3.58
ν	Mixture	p_1	p_2	p_3	RMSE	ν	Mixture	p_1	p_2	p_3	RMSE
0.5	1	1	0	0	3.58	0.7	1	1	0	0	3.58
	2	0	1	0	25.94		2	0	0	1	38.04
	3	0	0	1	38.04		3	0	0.2	0.8	28.77
	4	0.5	0	0.5	9.18		4	0	0.8	0.2	20.56
	5	0	0.5	0.5	18.35		5	0	0.2	0.8	28.77
	6	0.5	0.5	0	2.93		6	0.1	0.7	0.2	21.98
	7	0.5	0	0.5	9.18		7	0.1	0.9	0	18.24
	8	0	0.5	0.5	18.35		8	0.2	0.4	0.4	16.71
	9	0.5	0.5	0	2.93		9	0.4	0.4	0.2	6.63
	10	0.4	0.3	0.3	8.93		10	0.5	0	0.5	9.18
	11	0.3	0.4	0.3	11.5		11	0.7	0.2	0.1	2.03
	12	0.3	0.3	0.4	12.79		12	0.7	0.3	0	2.17
ν	Mixture	p_1	p_2	p_3	RMSE						
0.91	1	1	0	0	3.58						
	2	0	0	1	38.04						
	3	0	0.6	0.4	29.6						
	4	0	0.8	0.2	20.56						
	5	0	0	1	38.04						
	6	0.1	0	0.9	41.39						
	7	0.2	0.1	0.7	25.86						
	8	0.4	0.2	0.4	13.66						
	9	0.5	0.3	0.2	4.67						
	10	0.5	0.4	0.1	3.51						
	11	0.6	0.4	0	2.17						
	12	0.7	0	0.3	2.41						
